# Heterologous overexpression of the cyanobacterial alcohol dehydrogenase *sysr1* confers cold tolerance to the oleaginous alga *Nannochloropsis salina*

**DOI:** 10.3389/fpls.2023.1045917

**Published:** 2023-01-25

**Authors:** Jong-Min Lim, Sokyong Jung, Jae-Sun In, Youn-Il Park, Won-Joong Jeong

**Affiliations:** ^1^ Cell Factory Research Center, Korea Research Institute of Bioscience and Biotechnology, Daejeon, Republic of Korea; ^2^ Department of Biological Sciences, Chungnam National University, Daejeon, Republic of Korea

**Keywords:** tolerance to temperature stress, reactive oxygen species (ROS), antioxidant enzymes, alcohol dehydrogenase, *Nannochloropsis salina*

## Abstract

Temperature is an important regulator of growth in algae and other photosynthetic organisms. Temperatures above or below the optimal growth temperature could cause oxidative stress to algae through accumulation of oxidizing compounds such as reactive oxygen species (ROS). Thus, algal temperature stress tolerance could be attained by enhancing oxidative stress resistance. In plants, alcohol dehydrogenase (ADH) has been implicated in cold stress tolerance, eliciting a signal for the synthesis of antioxidant enzymes that counteract oxidative damage associated with several abiotic stresses. Little is known whether temperature stress could be alleviated by ADH in algae. Here, we generated transgenic lines of the unicellular oleaginous alga *Nannochloropsis salina* that heterologously expressed *sysr1*, which encodes ADH in the cyanobacterium *Synechocystis* sp. PCC 6906. To drive *sysr1* expression, the heat shock protein 70 (HSP70) promoter isolated from *N. salina* was used, as its transcript levels were significantly increased under either cold or heat stress growth conditions. When subjected to cold stress, transgenic *N. salina* cells were more cold-tolerant than wild-type cells, showing less ROS production but increased activity of antioxidant enzymes such as superoxide dismutase, ascorbate peroxidase, and catalase. Thus, we suggest that reinforcement of alcohol metabolism could be a target for genetic manipulation to endow algae with cold temperature stress tolerance.

## Introduction

Temperature is a key regulator of the growth and development of living organisms such as algae and plants. Each organism has its own optimal temperature range; temperatures outside of this range are detrimental to growth and development (Yadav, 2010; Suzuki et al., 2011; Hasanuzzaman et al., 2012a; Kumar et al., 2013a). Accumulation of reactive oxygen species (ROS) and increased expression of heat shock- and stress-related proteins are primary responses to temperature stress (Croser et al., 2003; Beck et al., 2007; Saidi et al., 2011; Cheng and He, 2014; Kobayashi et al., 2014). In algae, ROS can be generated in chloroplasts, peroxisomes, mitochondria, and cytosol. An imbalance between ROS generation and scavenging activity results in oxidative damage such as oxidation of proteins, DNA damage, the peroxidation of membrane lipids, as well as destruction of photosynthetic pigments (Apel and Hirt, 2004; Xu et al., 2006; Hasanuzzaman et al., 2012a; Rezayian et al., 2019).

In algae, as in other higher plants, ROS generation in chloroplasts occurs mostly in the thylakoid membrane during photosynthetic electron transport. Stromal side production of ROS is mediated by photosystem I-driven electron transport to molecular oxygen, yielding superoxide species. ROS, such as hydrogen peroxide and singlet oxygen, can also be generated from photosystem II when the rate of electron flow to the mobile electron transport carrier plastoquinone exceeds the rate of electron flow out of the plastoquinone pool to the cytochrome b_6_f complex, resulting in photoinhibition of photosynthetic apparatus, including photosystem II and I (Eberhard et al., 2008). Such high redox poise could be exacerbated by various abiotic and biotic stresses. For instance, a combination of chilling stress and illumination increases excitation pressure on photosystem II to a greater extent than conditions in which either light or low temperature stress is provided to algae and higher plants independently (Maxwell et al., 1995; Huner et al., 1998). Algae, like other organisms, have evolved enzymatic or non-enzymatic defense responses to minimize damage from ROS and maintain cellular homeostasis (Kotak et al., 2007). Superoxide dismutase (SOD), ascorbate peroxidase (APX), and catalase (CAT) are antioxidant enzymes. Non-enzymatic antioxidants include pigments such as carotenoids and anthocyanins, ascorbic acids, glutathione, and flavonoids (Rezayian et al., 2019). Accordingly, ROS-scavenging activities in plants and microalga are directly related to temperature stress tolerance. For instance, higher antioxidant enzyme activities correlate with an increased tolerance to either high- or low-temperature stress (Huang and Guo, 2005; Almeselmani et al., 2009; Xing et al., 2022). In addition to oxidative damage-causing ligands, ROS act as signaling molecules for the synthesis of antioxidant enzymes, including CAT, APX, and SOD (Alscher et al., 2002; Wahid et al., 2007; Suzuki et al., 2011). Thus, enzymatic regulation of steady-state ROS to maintain levels that are neither too low to induce the defense mechanism nor too high to induce oxidative stress inside cells could be a feasible way to obtain algae-tolerant temperature-stress-induced ROS.

Alcohol dehydrogenases (ADHs), members of the dehydrogenase enzyme superfamily, are widely distributed across all living organisms (Chase, 1999; Strommer, 2011). These enzymes catalyze interconversion between alcohols and aldehydes under anaerobic conditions (Höög et al., 2003; Thompson et al., 2007). In plants such as *Arabidopsis* (Ismond et al., 2003), rice (Matsumura et al., 1998), and maize (Johnson et al., 1994), ADH is essential for their ability to tolerate hypoxia, in which expression of ADH genes is strongly induced. Additionally, expression of the gene encoding ADH is induced by numerous other environmental stresses, including cold and osmotic stress (Christie et al., 1991; Conley et al., 1999), wounding (Kato-Noguchi, 2001), drought stress (Dolferus et al., 1994; Senthil-Kumar et al., 2010), and salinity stress (Manak et al., 2002; Sobhanian et al., 2010). Although the physiological function of the ADH genes that accumulate in response to these stressors is largely unknown, ADH-overexpressing plants become more tolerant to abiotic stresses (e.g., cold stress) than wild-type plants, and this has been attributed to ADH-induced ROS-scavenging enzymes (Yi et al., 2017; Su et al., 2020). It is not known whether such cold resistance in photosynthetic algae could be obtained through genetic manipulation of ADH-mediated alcohol metabolism.

*Nannochloropsis* is a genus of oleaginous microalgae. Members of this genus exhibit high photosynthetic efficiency and produce large amounts of lipids. In addition, a well-established genetic toolbox is available, and large-scale outdoor cultivation is feasible (Rodolfi et al., 2009; Radakovits et al., 2012; Vieler et al., 2012; Wang et al., 2016). The optimum temperature for *Nannochloropsis* sp. is 24–26°C (Yamasaki and Hirata, 1995; Abu-Rezq et al., 1999); hence, temperatures below 15°C (Gill et al., 2018) or above 32°C (Sukenik et al., 2009) are detrimental to growth and photosynthesis, although low temperature induces a metabolic shift to lipid accumulation (Gill et al., 2018). Unlike other common algae *Chlamydomonas* and cyanobacteria, *N. salina* is equipped with weak inorganic carbon-concentrating mechanisms due to a CO_2_-leaking ${\text{HCO}}_{3}^{-}$ pump, and a lack of a pyrenoid and CO_2_ channel (Vikramathithan et al., 2020).

This study was undertaken to evaluate whether temperature stress could be alleviated by manipulating the alcohol metabolism of *N. salina* through heterologous expression of *sysr1*, the gene encoding ADH in the unicellular cyanobacterium *Synechocystis* sp. PCC 6906. First, we investigated whether expression of ADH genes and ADH enzyme activities are subject to temperature stress under high CO_2_ conditions. Then, we explored the feasibility of manipulating *N. salina* tolerance to temperature stress by increasing ADH activity levels. We generated transgenic strains of *N. salina* that express *sysr1* in a temperature change-dependent manner using its own heat shock 70 promoter. The transgenic strains were more tolerant to cold stress than the wild-type strains. Thus, we suggest that manipulation of ADH activity during light-chilling stress is a genetic target for generating cold-tolerant *N. salina*.

## Materials and methods

### Culture conditions

*Nannochloropsis salina* CCMP1776 obtained from the National Center for Marine Algae and Microbiota (formerly the Culture Collection of Marine Phytoplankton, CCMP) was cultured in modified f/2N medium (Kilian et al., 2011) containing 15 g/L sea salt (Sigma-Aldrich, USA), 427.5 mg/L NaNO_3_, 30 mg/L NaH_2_PO_4_·2H_2_O, 5 mL/L trace metal mixture [consisting of Na_2_ EDTA·2H_2_O (4.36 g/L), FeCl_3_·6H_2_O (3.15 g/L), CoCl_2_·6H_2_O (10 mg/L), ZnSO4·7H_2_O (22 mg/L), MnCl_2_·4H_2_O (180 mg/L), CuSO_4_·5H_2_O (9.8 mg/L), and Na_2_MoO_4_·2H_2_O (6.3 mg/L)], and 2.5 mL/L vitamin stock [consisting of vitamin B_12_ (1 mg/L), biotin (1 mg/L), and thiamine·HCl (200 mg/L)], as well as 10 mM Tris-HCl to maintain pH 7.6. The cells were maintained in ambient air at 25°C under a light intensity of 120 μmol/m^2^/s.

### Generation of *sysr1*-expressing transgenic *N. salina*

Transgenic lines of *N. salina* heterologously expressing an ADH were generated using a cyanobacterial *sysr1* that encodes ADH from *Synechocystis* sp. PCC 6906, as *sysr1* has been successfully used to generate oxidative-stress-resistant *Arabidopsis* (Yi et al., 2017; Su et al., 2020). Gene synthesis was performed after codon optimization (**Supplementary Figure S1**). The synthetic *sysr1* gene was subcloned into a modified pNs201 vector using BspHI and EcoRV restriction enzymes to produce the transformation vector pNsSysr1. Then, transformation was carried out using a protocol described previously (Vikramathithan et al., 2020). Cells grown in f/2N liquid medium were harvested at the mid-exponential phase (4 × 10^8^ cells/mL), washed three times with fresh 375 mM sorbitol, and then resuspended in 375 mM sorbitol to a concentration of 5 × 10^9^ cells/mL. One hundred microliters of concentrated cells (i.e., 5 × 10^8^ cells) and 5 μg of XbaI-linearized pNsSysr1 vector were mixed in 2-mm cuvettes (BTX, USA) prior to transformation. Then, electroporation was performed with the prepared cells and DNA mixtures using an ECM 850 square wave electroporation system (BTX, MA, USA). The following conditions: 50 pulses, 100-μs pulses, 500-ms intervals, and a high-voltage field strength of 12,000 V cm^−1^. After electroporation, the cells were resuspended in 10 mL f/2N media and incubated for 1 day at 25°C in the dark. Cells were centrifuged at 1,000 x *g* for 5 min and then plated onto f/2N agar medium containing Zeocin (2.5 μg/mL).

### Growth under temperature stress

Cold- and high-temperature stress was imposed by incubating cells (5 × 10^7^ cells/mL) in liquid or agar solid media at either 4°C for 7 days or 37°C for 3 days (for liquid medium) or 0.5 days (for solid medium) under white light (120 μmol/m^2^/s), and supplied with high (2%) CO_2_. Then, temperature-stressed cultures were transferred to a growth chamber at 25°C with high CO_2_. For liquid cultures, absorbance (750 nm) of cells after 3 days of incubation was measured using a spectrophotometer (Shimadzu, Japan). Specific growth rates (µ) were calculated as follows: µ = ln(*x*_2_) - In(*x*_1_)/*t*_2_-*t*_1_, where µ is the specific growth rate and *x*_1_ and *x*_2_ represent the number of cells at time 1(*t*_1_) and time 2(*t*_2_), respectively. For the spot growth test, cells (7 × 10^4^ –7 × 10^6^) were inoculated onto the f/2N agar plates were transferred to temperature treatment conditions. Cell images were taken 14 days after incubation at 25°C. Light intensity and CO_2_ concentration during and after temperature treatments were kept the same.

### Quantitative real-time PCR (qRT-PCR)

For qRT-PCR, cDNAs were synthesized using total RNA with TOPscriptTM RT DryMIX (dT18) (Enzynomics, Daejeon, Korea), according to the manufacturer’s instructions. The qRT-PCR was performed using the CFX96 qPCR system (Bio-Rad) and TOPreal™ qPCR 2X PreMIX (SYBR Green with low ROX, Enzynomics, Daejeon, Korea). The *N. salina* gene encoding actin served as an internal control. The primers used for qRT-PCR are listed in the **Supplementary Table S1**. The qRT-PCR protocol was as follows: 95°C for 10 min; 40–50 cycles at 95°C for 5 s, and 56–57°C for 20 s. Relative quantification of gene expression was calculated using the Bio-Rad CFX Manager 3.1 program, with the following calculation formulae:

ΔCt = Target Ct mean – actin gene Ct,

ΔΔCt = Sample ΔCt mean - Reference ΔCt mean,

2^^(-ΔΔCt)^ = fold difference.

### Measurement of ADH and aldehyde dehydrogenase (ALDH) activity

The activities of ADH and ALDH were measured in cells from wild-type and transgenic lines (1 × 10^8^ cells) after temperature stress treatments. ADH activity was determined colorimetrically (TECAN^®^ SPARK, Austria) by quantifying the amount of NADH produced using an Alcohol Dehydrogenase Activity Colorimetric Assay Kit (Sigma-Aldrich). ALDH activity was determined colorimetrically (TECAN^®^ SPARK, Austria) by a coupled enzyme assay in which acetaldehyde was oxidized by ALDH generating NADH using an Aldehyde Dehydrogenase Activity Colorimetric Assay Kit (Sigma-Aldrich). An aliquot of cell extract (50 µL) was added to the assay medium before the assay and incubated for 1 h at 37°C or 25°C.

### Multiple alignments and phylogenetic tree

Multiple alignments with other known ADH proteins were performed using Clustal Omega. Phylogenetic analyses of putative *N. salina* ADH proteins were performed using MEGA11 with the neighbor-joining method (minimum evolution criterion and bootstrap values performed on 1000 replicates).

### Southern and Northern blot analyses

Genomic DNAs were extracted from wild-type and *sysr1*-expressing transgenic cells using a standard protocol (Lim et al., 2015). An aliquot (10 μg) of genomic DNA was digested with the *Kpn* 1 restriction enzyme, separated by electrophoresis on an 0.8% agarose gel, and blotted on a Hybond N^+^ nylon membrane (Amersham Biosciences, USA). A 0.3-kb PCR fragment corresponding to the C-terminal region of *sysr1* was used as a probe. Hybridization with ^32^P-labeled probes was performed in accordance with manufacturer’s instructions. The hybridization signals were detected using a Bio-Imaging Analyzer BAS-1800II (Fuji, Tokyo, Japan). For Northern blotting, total RNAs were isolated using Nucleozol (Macherey-Nagel, Germany), according to the manufacturer’s instructions. Total RNA (15 μg) was separated on a 1.2% agarose gel and then blotted onto a nylon membrane. The 0.3-kb *sysr1* fragment was used as a probe. The primer sequences used for the PCR probe in Southern and Northern blots are listed in the **Supplementary Table S1**.

### ROS measurement

Accumulation of ROS was assessed using a modified version of the method described by Halliwell and Gutteridge (1999). Briefly, cells were cultured under different conditions, harvested, and resuspended at a density of 1 × 10^7^ cells/mL in 2 mL of f/2N medium supplemented with 5 µM 2′,7′-dichlorofluorescein-diacetate (DCF-DA). Cells were then incubated in the dark at 25°C for 1 h, with gentle shaking. The fluorescence of the DCF-DA-treated cells was measured with a spectrofluorometer (Model LS55; PerkinElmer, Norwalk, CT, USA) at room temperature, with an excitation wavelength of 485 nm and an emission band between 500 and 600 nm. To determine relative ROS production, fluorescence intensity at 520 nm (F520) was used.

### Antioxidant enzyme activity assay

Enzyme activities were measured using a modified version of the method described by Elbaz et al. (2010). Briefly, cells (1 × 10^9^ cells) suspended in 1.5 mL of cold extraction buffer containing 50 mM Tris–HCl (pH 7.8), 1 mM EDTA, 1 mM MgCl_2_, and 1% (w/w) polyvinylpyrrolidone were disrupted by sonication on ice (3 cycles of 5 pulses, power 12, Fisher Scientific Sonic Dismembrator Model F60, Pittsburgh, PA). For the APX assay, this buffer also contained 1 mM ascorbate. After sonication, crude extract was centrifuged at 14,000 x g at 4°C for 10 min, and the supernatant was collected. Protein concentration was calculated using the Bradford method, using bovine serum albumin as a standard (Bradford, 1976).

SOD activity was measured as the inhibition of the photochemical reduction of nitro-blue tetrazolium (NBT) (Beauchamp and Fridovich, 1971). The reaction mixture contained 50 mM sodium phosphate buffer (pH 7.8), 10 mM methionine, 1.17 mM riboflavin, 56 mM NBT, and 50 μL of enzyme extract in a final volume of 3 mL. The absorbance of the solution was measured at 560 nm. One unit of SOD was defined as the amount of enzyme needed to inhibit 50% of the NBT reduction for 2 min under the assay conditions. SOD activity was expressed in U mg^-1^ of protein.

CAT activity was assayed spectrophotometrically using 990 μL of reaction mixture containing 50 mM sodium phosphate buffer (pH 7.0), 10 mM H_2_O_2_, and 10 μL of enzyme extract. The decrease in absorbance at 240 nm was measured for up to 2 min, and CAT activity was calculated using the extinction coefficient of 0.036 mM^−1^ cm^−1^ (Aebi, 1984).

APX activity was evaluated spectrophotometrically. The reaction mixture (990 μL) contained 50 mM sodium phosphate buffer (pH 7.0), 0.5 mM ascorbate, 0.1 mM H_2_O_2_, and 10 μL of the enzyme extract. APX activity was calculated from the change in absorbance at 290 nm due to ascorbate oxidation, and determined using an extinction coefficient of 2.8 mM^−1^ cm^−1^ (Nakano and Asada, 1981).

### Accession numbers

The gene sequences used in this study can be found in the NCBI database library under the following accession numbers: OP168497 (NsADH1), OP168498 (NsADH2), OP168499 (NsADH3), OP168500 (NsALDH1), OP168501 (NsALDH2), OP168502 (NsHSF), OP168503 (NsHSP70), OP168504 (NsHSP90), OP168505 (NsHSP100), OP168506 (NsAPX), OP168507 (NsSOD), OP168508 (NsCAT), and OP168509 (NsACT1).

### Statistical analysis

Statistical analysis was conducted using one-way ANOVA. Data are expressed as the mean ± SE, and *p* < 0.05 was considered statistically significant. Different letters indicate a significant difference.

## Results

### Temperature stress reduces the transcript level and activity of ADH in *N. salina*

**Figure 1:**
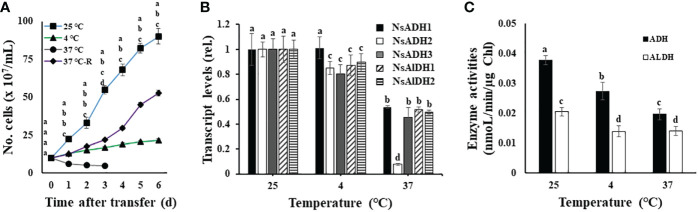
Temperature-dependent growth **(A)**, transcript levels **(B)**, and enzyme activities **(C)**, of alcohol dehydrogenase (ADH) and aldehyde dehydrogenase (ALDH) in wild-type *N. salina* cells. **(A)** Cells from the mid-exponential growth phase grown at 25°C were subjected to either cold (4°C, 7 days) or heat (37°C, 0.5 days (37°C-R) or 7 days) stresses. After temperature stress treatments, cells were transferred to 25°C and then incubated for 6 days. **(B, C)** ADH and ALDH transcript abundance **(B)** and enzyme activity **(C)** in *N. salina* cells exposed to optimal, low, or high temperatures for 3 days, 7 days, or 0.5 days, respectively, and collected. Temperature stress was provided under illumination (120 μmol/m^2^/s^1^) in the presence of 2% CO_2_. Data are expressed as the mean ± SE; n = 5. Means denoted by different letters indicate a significant difference (one-way ANOVA; *p* < 0.05).

Currently, we do not know whether temperature stress-induced growth disturbance is related to ADH activity in *N. salina* cells. To set up temperatures below or above optimal growth, *N. salina* cells were grown under room (25°C), low (4°C), and high (37°C) temperature growth conditions. Cells were cultured for 6 days under continuous white light illumination (120 μmol/m^2^/s) with 2% CO_2_. A high CO_2_ concentration and continuous illumination were maintained throughout temperature stress treatments to avoid disturbance of photosynthetic electron transport due to limited CO_2_. Thus, under such conditions, responses of *N. salina* to temperature changes are highly related to the effects of temperature on light-driven photosynthetic electron transport activities. Consistent with previous reports (Yamasaki and Hirata, 1995; Abu-Rezq et al., 1999; Sukenik et al., 2009), cells grew more slowly at low or high temperatures than at room temperature (**Figure 1A**), indicating that *N. salina* cells are subjected to either light-chilling or light-warming stress.

Next, we investigated whether such temperature stress affects ADH and ALDH expression (both of which are involved in fermentation metabolism) in *N. salina*. ADH and ALDH transcript levels were significantly lower in temperature-stressed cells than in non-stressed (control) cells (**Figure 1B**). ADH and ALDH transcript levels in cold-stressed cells decreased by 10–20%. Additionally, reduced transcript levels for both genes were observed in cells under heat stress (decreases of 46–92% and 48–50% for ADH and ALDH, respectively). Consistent with the decline in mRNA transcripts, the activity of these two enzymes was also lower under temperature stress than in the control optimal growth temperature conditions. The activity of ADH and ALDH decreased to 72.4% and 68.2% at 4°C, and 51.9% and 68.4% at 37°C, respectively (**Figure 1C**). This downregulation of ADH activity by temperature stress differs from higher plants, in which these enzymes are upregulated in response to similar temperature stress (Christie et al., 1991; Conley et al., 1999; Yi et al., 2017). Thus, ADH-dependent resistance to cold stress seems to be absent in *N. salina*.

### Generation of *sysr1*-expressing *N. salina* transgenic cells

**Figure 2:**
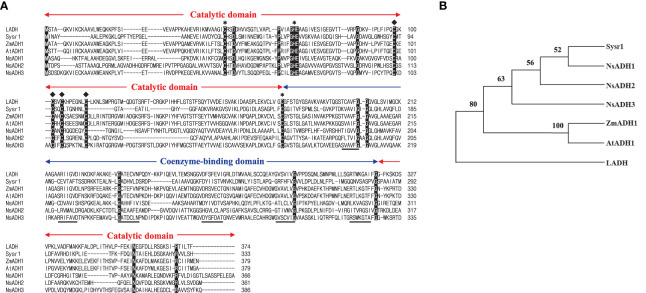
Amino acid sequence alignment **(A)** and phylogenetic tree **(B)** of selected alcohol dehydrogenases (ADHs). **(A)** Alignment of ADHs from horse liver (LADH), *Synechocystis* sp. PCC 6906 (sysr1), *Zea mays* (ZmADH1), *Arabidopsis thaliana* (AtADH1), and *Nannochloropsis salina* (NsADH1, 2, and 3). Conserved amino acids among all seven ADHs are marked in black. Catalytic and coenzyme-binding domains are indicated, along with the binding sites for the catalytic (**∗**) and structural (♦) Zn ions. The β sheets of the NAD-binding Rossmann fold (Rossmann et al., 1974) are underlined. **(B)** Phylogenetic tree of LADH (GenBank ID, KY014075), Sysr1 (NP_001075414.1), ZmADH1 (NP_001105409.2), AtADH1(NP_177837.1), NsADH1(UX097916.1), NsADH2(UX097917.1), and NsADH3(UX097918.1), generated using MEGA version 11.

**Figure 3:**
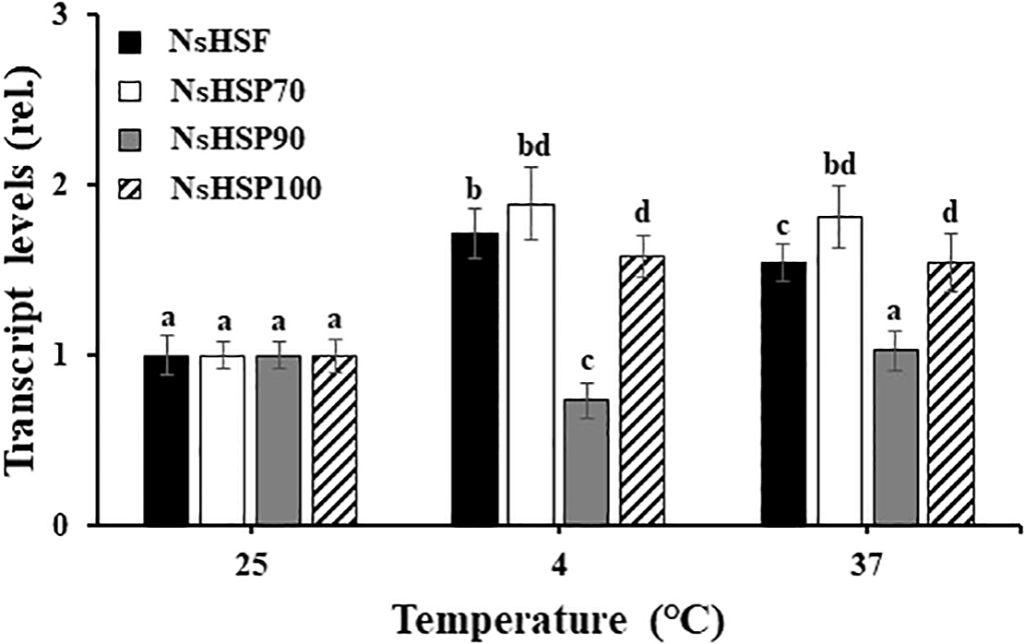
Transcript levels of genes encoding a heat shock transcription factor (HSF) and heat shock proteins (HSPs) in wild-type *N. salina* cells subjected to cold (4°C) or heat (37°C) stress for 7 or 0.5 days, respectively, under illumination (120 μmol/m^2^/s^1^) in the presence of 2% CO_2_. Data are expressed as the mean ± SE (n = 4). Means denoted by different letters indicate a significant difference (one-way ANOVA; *p* < 0.05).

**Figure 4:**
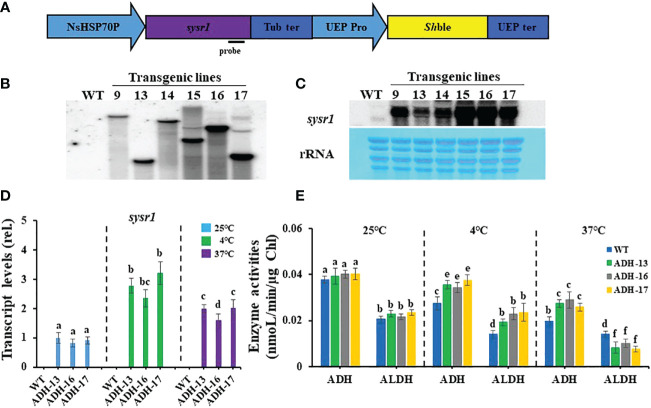
Generation of *sysr1*-expressing transgenic lines (**A**, **B**, **C**), *sysr1* transcript levels **(D)**, and enzyme activity of alcohol dehydrogenase (ADH) and aldehyde dehydrogenase (ALDH) **(E)**, in wild-type *N. salina* (WT) and transgenic cell lines (ADH-13, ADH-16, and ADH-17). **(A)** Schematic diagram of the pNsSysr1 vector. **(B)** Image of a Southern blot of transgenic lines. **(C)** Northern blot analysis of transgenic lines. Upper: Northern blot image. Lower: Methylene blue staining image of rRNA served as loading controls. **(D)** Transcript levels of the genes in wild-type *N. salina* and *sysr1*-overexpressing cells subjected to cold (4°C) or heat (37°C) stress for 7 or 0.5 days, respectively. **(E)** ADH and ALDH activity in cells subjected to cold (4°C) or heat (37°C) stress for 7 or 0.5 days, respectively. Temperature stress was generated under illumination (120 μmol/m^2^/s^1^) in the presence of 2% CO_2_. Data are expressed as the mean ± SE (n = 3). Means denoted by different letters indicate a significant difference (one-way ANOVA; *p* < 0.05). NsHSP70P, the promoter of the heat shock protein (HSP) 70 gene in *N. salina*; Tub ter, terminator of the β-tubulin-coding gene in *N. salina*; UEP pro and UEP ter; promoter and terminator of the ubiquitin extension gene in *N. salina*; *Sh*ble, Zeocin resistance gene.

To address whether *N. salina* cells with robust heterologous expression of ADH were more tolerant to temperature stress than wild-type *N. salina* cells, *sysr1*-expressing *N. salina* transgenic lines were generated. Rather than using the actual *N. salina* ADH gene as a homologous alternative, we searched for a heterologous ADH gene for transgenic construction (Park et al., 2009). Multiple sequence alignments and phylogenetic analysis showed that ADHs of *N. salina* are more closely related to cyanobacterial ADH genes (**Figure 2**), supporting its usefulness as a heterologous gene, as described previously (Yi et al., 2017). When generating *sysr1*-expressing transgenic *N. salina* cells, *sysr1* was placed under the control of the endogenous heat shock protein 70 (HSP70) promoter of *N. salina* rather than a constitutive promoter such as actin, so that the gene would be induced mostly under temperature-stress conditions. Among HSP and heat shock transcription factor (HSF) promoters, the HSP70 promoter was chosen rather than other heat shock transcription factors (HSF) or HSP90 and HSP100, as in contrast to these, transcript levels of HSP70 increased by ~1.9-fold under both temperature-stress conditions (**Figure 3**). Transgenics exhibiting temperature-inducible ADH expression, but with rather weak strength, avoid detrimental ethanol- or aldehyde-induced effects to cells that are highly likely when ADH expression is driven by a strong constitutive promoter. Insertion of the *sysr1* gene (**Figure 4A**) into the *N. salina* genome was confirmed by Southern blotting (**Figure 4B**), and high expression of the *sysr1* gene was confirmed by northern blotting (**Figure 4C**). Three transgenic lines, referred to as ADH13, ADH16, and ADH17, were selected for the rest of the study. Consistent with the 2–3-fold increases in *sysr1* transcript levels (**Figure 4D**), these lines exhibited ~1.3–1.7-fold higher ADH activity than wild-type cells at 4°C and 37°C. However, ALDH activity at 37°C was 1.3–1.7-fold lower than that in wild-type cells (**Figure 4E**).

### Heterologous *sysr1* expression increases cold stress tolerance in *N. salina*

**Figure 5:**
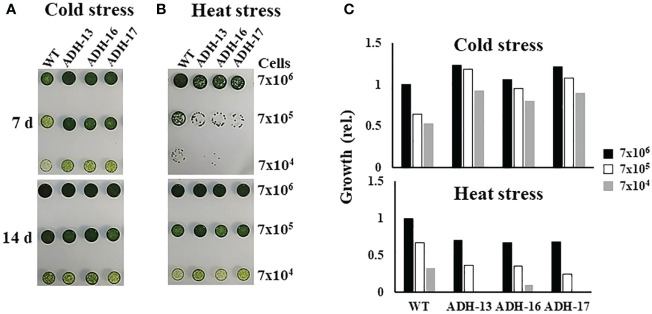
Growth of wild-type (WT) and *sysr1*-expressing transgenic *N. salina* under cold or heat stress. **(A)** Cold-stressed and control cells. **(B)** Heat-stressed and control cells. In **(A, B)**, the upper and lower panels show images of temperature-stressed or non-stressed control cells, respectively. **(C)** Spot densities of cold-stressed **(A)** and heat-stressed **(B)** cells. Cell droplets with low (7 × 10^4^), intermediate (7 × 10^5^), and high (7 × 10^6^) cell density were spotted onto f/2N agar medium and then subjected to cold stress at 4°C for 7 days, or heat stress at 37°C for 0.5 days, under illumination (120 μmol/m^2^/s^1^) in the presence of 2% CO_2_. Images were taken 7 or 14 days after temperature stress treatment. Spot densities were quantified and are shown as relative abundance normalized to the high (7 × 10^6^) density of respective cold and heat stress-treated wild-type cells, which is set as 1.0.

**Figure 6:**
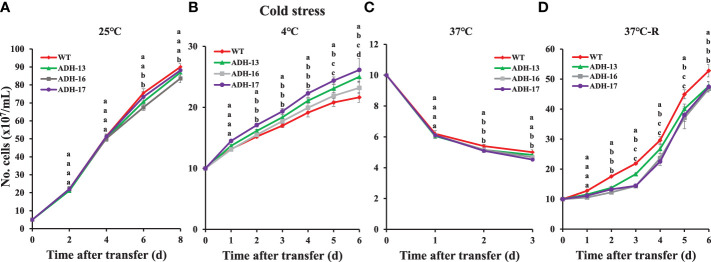
Temperature-dependent growth of wild-type (WT) and s*ysr1*-expressing cells of *N. salina*. **(A)** Control (25°C). **(B)** Continuous 4°C. **(C)** Continuous 37°C. **(D)** Incubated at 25°C after treatment at 37°C for 12 h. Under continuous illumination (120 μmol/m^2^/s^1^), cells were grown under optimal (25°C; **A**), cold (4°C; **B**), and heat (37°C; **C**) or heat shock (37°C for 12 h; **D**) stress conditions in the presence of 2% CO_2_. In **(D)**, cells were transferred to optimal temperature growth conditions after heat shock treatment. Data are expressed as the mean ± SE (n = 3); transgenic lines are identified in the legend. Means denoted by different letters indicate a significant difference (one-way ANOVA; *p* < 0.05).

Using *sysr1*-expressing *N. salina* lines, a spot growth test was carried out to investigate whether transgenic cell lines with higher ADH enzyme activities under either low or high temperature conditions are more tolerant to temperature stress than wild-type cells. As shown in **Figure 5**, cells grown at room temperature were plated onto agar medium, incubated with 2% CO_2_ either at 4°C for 7 days (**Figure 5A**, upper panel) or at 37°C for 0.5 days (**Figure 5B**, upper panel), and then transferred to 25°C and incubated for 14 days with 2% CO_2_. The wild-type and transgenic lines cultured at 25°C served as the controls (**Figures 5A, B**; lower panels). Cold-stressed transgenic lines clearly grew faster than the cold-stressed wild-type cells (**Figures 5A, C**; upper panel). In contrast to cold stress, all transgenic lines were more sensitive to heat stress, showing strongly retarded growth (**Figures 5B, C**; lower panel). These opposing responses of transgenic lines to cold and heat stress was also apparent when growth was assessed in liquid culture. When cultured in f/2N liquid medium at 37°C, the transgenic lines showed severe growth defects, while those at 4°C were more cold-tolerant than the wild type (**Figure 6**).

### Heterologous *sysr1* expression reduces ROS accumulation in *N. salina* under cold stress

**Figure 7:**
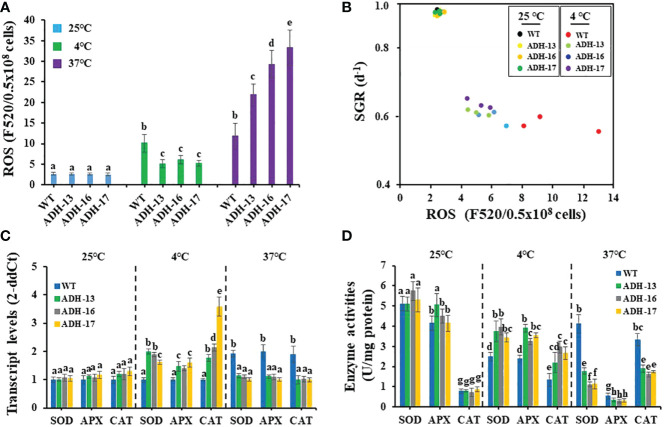
Reactive oxygen species (ROS) content **(A)**, cell growth versus ROS content **(B)**, transcript levels **(C)**, and enzyme activity **(D)** of SOD, APX, and CAT in wild-type (WT) and s*ysr1*-expressing *N. salina.* **(A)** ROS content in wild-type and transgenic cells subjected to cold or heat stress under continuous illumination (120 μmol/m^2^/s^1^) in the presence of 2% CO_2_. **(B)** Relationship between ROS contents and specific growth rate (SGR) calculated from **Figure 5**. **(C)** Relative expression level of genes encoding SOD, APX and CAT. **(D)** Antioxidant enzyme activities of SOD, APX, and CAT. *N. salina* cells subjected to different temperature conditions (25°C for 3 days, 4°C for 7 days, or 37°C for 0.5 days) under continuous illumination (120 μmol/m^2^/s^1^) in the presence of 2% CO_2_. Data are expressed as the mean ± SE (n = 3). Means denoted by different letters indicate a significant difference (one-way ANOVA; *p* < 0.05).

The detrimental effects of temperature stress on the growth of other algae and plants are related to ROS accumulation (Shi et al., 2017), and this led us to investigate whether *N. salina*, when subjected to temperature stress, exhibited a ROS burst. Under optimal growth temperature, the ROS content of cells of the transgenic lines was ~1.2–5.9% less than that of wild-type cells (**Figure 7A**). As with other oleaginous microorganisms, *N. salina* also exhibited a 3.8-fold increase in ROS content upon exposure to cold stress. As expected, transgenic cell lines showed only a ~2.3-fold increase in ROS content compared with those at optimal growth temperature (**Figure 7A**). Heat stress also induced ROS accumulation in both wild-type and transgenic cell lines. *N. salina* exhibited a 4.5-fold increase in ROS content upon exposure to heat stress. However, in contrast to cold stress, transgenic lines showed a ~1.9–2.8-fold increase in ROS compared with the wild-type control (**Figure 7A**). As ROS levels were inversely related to cell growth (**Figure 7B**), temperature stress seems to impair algal growth *via* ROS-mediated oxidative damage.

### Heterologous *sysr1* expression increases antioxidant enzyme activity in *N. salina*

Antioxidant enzymes activities under ROS burst were investigated in temperature-stressed *N. salina* transgenic lines. Transcript levels and activities of three antioxidant enzymes (SOD, APX, and CAT) were measured in *N. salina* wild-type and transgenic lines. At normal temperatures, expression of the genes encoding these enzymes was slightly higher in the transgenic lines than in the wild-type, whereas under cold stress, levels were significantly greater in the transgenic lines than in the wild-type. These increases were 1.4–1.6-fold for APX, 1.6–2.0-fold for SOD, and 1.8–3.6-fold for CAT (**Figure 7C**). Under heat stress, the transcript levels of the genes encoding these antioxidant enzymes were lower in the transgenic lines than in wild-type cells (**Figure 7C**).

Next, we measured the enzyme activity of SOD, APX, and CAT in wild-type and transgenic control and temperature-stressed cells (**Figure 7D**). Enzyme activity in transgenic and wild-type cells was similar under optimal temperatures, but greater in cold-stressed transgenic cells than in the wild-type. SOD, APX, and CAT activity increased by 1.4–1.6-fold, 1.4–1.6-fold, and 1.6–2.2-fold, respectively. However, under heat stress, SOD, APX, and CAT activity was significantly lower in transgenic lines than in the wild type. SOD, APX, and CAT activity decreased to 26.9–43.0%, 52.6–62.4%, and 48.5–57.2%, respectively, in transgenic lines compared with the wild-type (**Figure 7D**). We suggest that, in transgenic lines, sysr1 induced antioxidant enzyme activity during cold stress, and this in turn reduced ROS levels. However, by contrast, this protein reduced SOD, APX, and CAT activity during heat stress, permitting ROS to accumulate more than in the wild-type.

## Discussion

The ADH-dependent ROS defense mechanism operates in plants, where its expression can be induced by cold (Christie et al., 1991; Conley et al., 1999). However, this scenario is not applicable to *N. salina*. Despite the presence of three ADH (ADH1, ADH2, and ADH3) and two ALDH (ALDH1 and ALDH2) genes in the genome, their transcript levels and enzyme activities were downregulated during temperature stress (**Figure 1C**). Thus, *N. salina* seems to lack ADH-dependent antioxidant activity for coping with temperature stress.

We observed that the detrimental growth of *N. salina* caused by cold stress was rescued by heterologous ADH expression. Under optimal growth temperatures, there is a dynamic equilibrium between generation of ROS and scavenging capacity. Thus, temperatures above or below the optimal temperature may disturb this balance, leading to oxidative stress, as has been noted for other microorganisms (Shi et al., 2017). Consistent with this, growth inhibition in *N. salina* during temperature stress is strongly related to ROS accumulation, which is in turn caused by reduced expression of both ADH and antioxidant scavenging enzymes (**Figure 7**). When *sysr1* was induced by low temperature cue, these enzyme activities decreased to a lesser extent than those of wild-type lines due to increased transcript accumulation of these genes. Accordingly, transgenic lines became more cold-tolerant in terms of growth performance.

Currently, we do not fully understand the mechanism by which heterologous expression of cyanobacterial ADH maintains the resistance of *N. salina* cells to low temperature stress. Abundant ROS content in low temperature-stressed *N. salina* seems to be related to reduced activity of antioxidant enzymes such as SOD, APX, and CAT (**Figure 7**). Consistent with this view, in transgenic cells with lowered ROS content by *sysr1*-expression, these enzyme activities remained higher than those in wild-type cells. Therefore, it is highly likely that ADH enzyme activity somehow regulates antioxidant enzyme activity, presumably at the transcriptional level. It is known that alcohol promotes ROS production in both a cytochrome P450- and Fenton reaction-dependent manner (Wu and Cederbaum, 2003); thus, if we consider ROS as signaling molecules (Alscher et al., 2002; Wahid et al., 2007; Suzuki et al., 2011), then ROS produced by ethanol could negatively affect expression of the SOD, APX, and CAT genes in *N. salina*.

It is noteworthy that the ROS content in heat-stressed transgenic lines was 1.9–2.8-fold higher than that in wild-type cells, suggesting significantly lower scavenging enzyme activity than that in optimal or chilling-stressed cell lines. Both formation and degradation of acetaldehyde that is the main toxic metabolite of alcohol and induces ROS generation depends on the activity of ADH and ALDH enzymes (Yan and Zhao, 2020). Accordingly, such a high ROS content might result from acetaldehyde accumulation due to lowered ALDH activity (**Figure 4E**), which overwhelms ROS-scavenging enzyme activity. This kind of imbalance between ROS production and antioxidant protective function leads to impaired growth performance under heat stress. In addition, non-enzymatic antioxidant levels could be lowered by heat stress, as their levels are prone to decline upon exposure to alcohol (Wu and Cederbaum, 2003).

In addition to biomass production, oil accumulation and fatty acid composition of algae are affected by temperature, as well as other environmental factors such as light intensity, salinity, low oxygen, and dehydration (Vitova et al., 2015; Shi et al., 2017). Indeed, in *Nannochloropsis oculata* and *N. salina*, warming and cooling induces changes in total lipid accumulation and lipid composition (Converti et al., 2009; Gill et al., 2018). It will be of interest to determine whether temperature-sensitive changes in lipid production and composition are related to ADH-dependent ROS metabolism.

To summarize, we expressed *sysr1*, an ADH gene from *Synechocystis* sp. PCC 6906, in the microalga *N. salina*. *N. salina* expressing *sysr1* was cold-tolerant but heat-sensitive. Acquired cold tolerance is attributable to the modulation of ROS balance, favoring reinforcement of ROS-scavenging activities. Response to heat stress seems to originate from an ADH-dependent ROS burst that eventually leads to cellular damage, resulting in retarded cell growth. Fine-tuning of alcohol metabolism, which is sensitive to temperature changes, would be a challenging target for genetic manipulation to confer temperature stress tolerance on algae.

## Data availability statement

The datasets presented in this study can be found in online repositories. The names of the repository/repositories and accession number(s) can be found in the article/**Supplementary Material**.

## Author contributions

W-JJ designed the research. J-ML, SJ, and J-SI conducted the experiments. W-JJ and Y-IP analyzed the data. J-ML, Y-IP, and W-JJ wrote the manuscript. All authors contributed to the article and approved the submitted version.
