# Supplementary material for: Heterologous overexpression of the cyanobacterial alcohol dehydrogenase sysr1 confers cold tolerance to the oleaginous alga Nannochloropsis salina
Source: Front Plant Sci. 2023 Jan 25;14:1045917. doi: 10.3389/fpls.2023.1045917 (PMC9905847; doi:10.3389/fpls.2023.1045917)
Supplement: Supplementary file 1 [file DataSheet_1.docx]

ATGATCAACGCCTACGCTGCCCTGGAGCCCAAGGGCAAGCTCCAGCCCTTCACCTACGAGCCTGGCGAGCTGGCTGCCAACGAGGTGGAGATCGCCGTGGACTACTGCGGCCTGTGCCACTCCGACCTGTCCATGATCAACAACGAGTGGGGCATCACCGCCTACCCGCTGGTGCCTGGCCACGAGGTGGTGGGCAAGGTGGCTGCCATCGGCGACCAGATCTCCCACCTGGCCGTGGGCGACGCCGTGGGCCTGGGCTGGCACTCCGGCTACTGCATGACCTGCTCCCAGTGCCTGACCGGCAACCACAACCTGTGCGGCACCGCCGAGGCCACCATCCTGGGCCACTACGGTGGCTTCGCCGACAAGGTGCGCGCCAAGGGCGTGTCCGTGATCAAGCTGCCCGACGGCATCGACCTGGCCTCCGCTGGTCCCCTGTTCTGCGGTGGCATCACCGTGTTCTCGCCCATGCTGTCCCTGGGCGTGAAGCCCACCGACAAGGTGGCCGTGATCGGCATCGGTGGCCTGGGCCACCTGGCCGTGCAGTTCCTGGACGCCTGGGGCTGCGAGGTGACCGCCTTCACCTCCTCCGACCGCAAGAAGACCGAGGCCCTGAACCTGGGTGCTGACCACGTGCTGGACTCCCGCGACCCGGAGGCCATCGCTGCCGTGCAGGGCAAGTTCGACTACATCATCTCCACCGTGAACGTGAAGCTGGACTGGAACCTGTACATCTCCACCCTGGCTCCCCAGGGTCGCTTCCACTTCGTGGGCGTGGTGCTGGACCCGCTGGACCTGAACCTGTTCCCTCTGATCATGGGCCAGCAGAACGTGTCCGCCTCGCCCGTGGGCTCGCCTGCTGCCATCGCCACCATGCTGGACTTCGCCGTGCGCCACGACATCAAGCCGCTGATCGAGACCTTCAAGTTCGACCAGATCAACACCGCCATCGAGCACCTGGACTCCGGCAAGGCTCACTACCGCGTGGTGCTGTCCCACTGA

**Supplementary Figure S1.** Codon-optimized nucleotide sequence of *sysr1* gene.
