# Supplementary material for: Heterologous overexpression of the cyanobacterial alcohol dehydrogenase sysr1 confers cold tolerance to the oleaginous alga Nannochloropsis salina
Source: Front Plant Sci. 2023 Jan 25;14:1045917. doi: 10.3389/fpls.2023.1045917 (PMC9905847; doi:10.3389/fpls.2023.1045917)
Supplement: Supplementary file 2 [file Table_1.docx]

**Supplementary Table S1.** Sequences of the primers used in this study

| **Gene** | **Primer** | | **Sequence 5’–3’** | **Purpose** |
| --- | --- | --- | --- | --- |
| sysr1 | | Sysr1-F1 | AGGTGACCGCCTTCACCTCCT | Southern and Northern blot probe |
|  |  | Sysr1-R1 | ATCGCCACCATGCTGGACTTCG |  |
| NsADH1 | | NsADH1-F1 | GCGCGCACCATTTCATCGTG | qRT-PCR |
|  |  | NsADH1-R1 | ACCTTGAGGCGCTGCATGAG |  |
| NsADH2 | | NsADH1-F1 | GGTGGTGGACCAGGTCATTGC | qRT-PCR |
|  |  | NsADH1-R1 | AACAATGCTCCCTCGCACGG |  |
| NsADH3 | | NsADH1-F1 | GACAAGCCCATTCAGCAGGTGA | qRT-PCR |
|  |  | NsADH1-R1 | CCGAATGCCGTGCCTTTCCA |  |
| NsALDH1 | | NsALDH1-F1 | GGCGCGAGTGGAAGGCATCAAT | qRT-PCR |
|  |  | NsALDH1-R1 | GTTGCTCTGTCACACGCTTCAGCT |  |
| NsALDH2 | | NsALDH2-F1 | GCACCTGGCCTCCGGATTCTTCTA | qRT-PCR |
|  |  | NsALDH2-R1 | GGTGCGTTTCGATGGTGCTCGTT |  |
| NsHSF | | NsHSF-F1 | CAGCCATTGTCACCAGGCGT | qRT-PCR |
|  |  | NsHSF-R1 | CCCATGCTCAGCTTCTCGACCT |  |
| NsHSP70 | | NsHSP70-F1 | TCCCATCCAAGTGCGGATGGA | qRT-PCR |
|  |  | NsHSP70-R1 | CTGGATCCACGTGGGACGGTA |  |
| NsHSP90 | | NsHSP90-F1 | TATGGAGCGCATCATGCGCA | qRT-PCR |
|  |  | NsHSP90-R1 | ACGTCATCGTGGGTGAAGCCT |  |
| NsHSP100 | | NsHSP100-F1 | AGGATCGTCTTCGCCAGCACT | qRT-PCR |
|  |  | NsHSP100-R1 | GGCACCGTTTGCCACGTCATA |  |
| NsSOD | | NsSOD-F1 | GCCTGGCTGGTCCTCAAGGA | qRT-PCR |
|  |  | NsSOD-R1 | GACCACCAGGCCTCCACGTA |  |
| NsAPX | | NsAPX-F1 | CAGGGAGGCTGTCCTCCTGT | qRT-PCR |
|  |  | NsAPX-R1 | AGAGCCCAAACCGACCGTCT |  |
| NsCAT | | NsCAT-F1 | GCGCCCAACTACTTCCCCAAT | qRT-PCR |
|  |  | NsCAT-R1 | AAAGTTGGCGACGGCACGTT |  |
| NsAct | | NsAct-F1 | GGTCTCGTACTTCGGTGACAAGGA | qRT-PCR |
|  |  | NsAct-R1 | ACGTGTTGGTCGCCAATGGT |  |
